# Optimization of photochemical decomposition acetamiprid pesticide from aqueous solutions and effluent toxicity assessment by *Pseudomonas aeruginosa* BCRC using response surface methodology

**DOI:** 10.1186/s13568-017-0455-5

**Published:** 2017-08-04

**Authors:** Ali Toolabi, Mohammad Malakootian, Mohammad Taghi Ghaneian, Ali Esrafili, Mohammad Hassan Ehrampoush, Maesome Tabatabaei, Mohsen AskarShahi

**Affiliations:** 1 Environmental Science and Technology Research Center, Department of Environmental Health Engineering, Shahid Sadoughi University of Medical Sciences, Yazd, Iran; 2 Environmental Health Engineering Research Center, Kerman University of Medical Sciences, Kerman, Iran; 3 Department of Environmental Health, School of Public Health, Kerman University of Medical Sciences, Kerman, Iran; 4 Department of Environmental Health Engineering, School of Public Health, Iran University of Medical Sciences, Tehran, Iran; 5 Department of Chemistry, Islamic Azad University, Yazd, Iran; 6 Department of Biostatistics and Epidemiology, Shahid Sadoughi University of Medical Science, Yazd, Iran

**Keywords:** Photocatalytic decomposition, Acetamiprid, Toxicity assessment, *Pseudomonas aeruginosa* BCRC

## Abstract

Contamination of water resources by acetamiprid pesticide is considered one of the main environmental problems. The aim of this study was the optimization of acetamiprid removal from aqueous solutions by TiO_2_/Fe_3_O_4_/SiO_2_ nanocomposite using the response surface methodology (RSM) with toxicity assessment by *Pseudomonas aeruginosa* BCRC. To obtain the optimum condition for acetamiprid degradation using RSM and central composite design (CCD). The magnetic TiO_2_/Fe_3_O_4_/SiO_2_ nanocomposite was synthesized using co-precipitation and sol–gel methods. The surface morphology of the nanocomposite and magnetic properties of the as-synthesized Fe_3_O_4_ nanoparticles were characterised by scanning electron microscope and vibrating sample magnetometer, respectively. In this study, toxicity assessment tests have been carried out by determining the activity of dehydrogenase enzyme reducing Resazurin (RR) and colony forming unit (CFU) methods. According to CCD, quadratic optimal model with R^2^ = 0.99 was used. By analysis of variance, the most effective values of each factor were determined in each experiment. According to the results, the most optimal conditions for removal efficiency of acetamiprid (pH = 7.5, contact time = 65 min, and dose of nanoparticle 550 mg/L) was obtained at 76.55%. Effect concentration (EC_50_) for RR and CFU test were 1.950 and 2.050 mg/L, respectively. Based on the results obtained from the model, predicted response values showed high congruence with actual response values. And, the model was suitable for the experiment’s design conditions.

## Introduction

Due to the ever-increasing growth of population and increased need for agricultural crops, food and fight with pathogenic carriers, use of pesticides has also grown in various sectors. Contamination of water with pesticides is usually caused by agricultural runoffs and the wastewater of toxin-producing industries. The flow of surface water owing to rainfall or irrigation of farmlands carries these materials and introduces them to rivulets and rivers (Samadi et al. 2010; Akhlaghian and Sohrabi 2015; Hossain et al. 2015; Ryberg and Gilliomb 2015; Stehle and Schulz 2015). Today, different pesticides are used for fighting vectors. Acetamiprid is a systemic, contact, and digestive insecticide, belonging to a new class of neonicotinoid insecticides, which is considered one of the most important micro-contaminants. Its solubility in water is 4250 mg/L at 25 °C and has a half-life of 5–15 days in the environment (Fasnabi et al. 2012; Carra et al. 2015). Acetamiprid is widely used in controlling pests of agricultural crops (John et al. 2016). However, most of the time due to unfamiliarity of chemical toxins, consumers are unaware about the damaging effects of these toxins and correct fighting principles. Due to high solubility, stability and presence of some resistant compounds in the structure of acetamiprid, conventional water and wastewater treatment technologies are not effective in its removal (Miguel et al. 2012; Mutsee 2013; Shanping et al. 2014; Sahithya and Das 2015; Jafari et al. 2014).

Some studies have been conducted for removing acetamiprid pesticide. John et al. (2016) in Kerala by coagulation and flocculation process (John et al. 2016). Wang (2013) in China used biological processes for removing acetamiprid (Wang et al. 2013). Similarly, Shanping (2014) and Fasnabi (2012) employed low-temperature heat plasma and ozonation process, respectively for removal of acetamiprid (Fasnabi et al. 2012; Shanping et al. 2014).

Recently, advanced oxidation processes (AOP) have attracted a great deal of attention, thanks to chemical stability, recoverability, low costs, and processing routes for detoxifying contaminants. They can be a potential substitute for physical, chemical, and biological methods of toxin removal (Ahmed et al. 2011; Moussavi et al. 2014).

TiO_2_ nanoparticles along with UV radiation are considered a suitable method in water and wastewater treatment (Mohammadi and Sabbaghi 2014; Sivashankar et al. 2014). However, as the distance of energy balances of TiO_2_ is lower than 3.2 eV, thus it receives only a small part of the ray spectrum and uses it for photocatalytic activity. Accordingly, to enhance optimal response, some changes should be made in its structure. With this aim in mind, for full employment of UV radiation and improving photocatalytic activity, doping of TiO_2_ nanoparticles with magnet, silica, carbon, and silver-containing materials can be of benefit (Samadi et al. 2010; Liu 2012; Wang et al. 2012; Cao et al. 2013).

Therefore, in the current study, SiO_2_ and Fe_3_O_4_ were introduced to the reaction. Introduction of magnetic nanoparticles into the TiO_2_ matrices resulted in prevention of accumulation of nanoparticles, catalyst durability, diminished cavity among nanoparticles, increased surface of the catalyst, and recoverability. Since silica particles are hydrophilic, due to presence of silanol group, activity of the surface of nanoparticles increased (Wu et al. 2009; Miguel et al. 2012; Shi et al. 2012; Wang et al. 2012; Cao et al. 2013).

Response surface methodology has been found to be a useful tool to study the interactions of two or more variables. RSM is a collection of mathematical and statistical techniques which are used considering several affecting factors in an optimum manner, even in the presence of complex interactions. It also gives a lot of information from a small number of experiments compared to conventional methods (Sahoo and Gupta 2012; Salman 2014; Mansouriieh et al. 2015; Yousefi et al. 2015). In this study, the photo catalytic experiments were designed based on CCD model for variables such as contact time, initial concentration of acetamiprid, dose of nanoparticles and pH.

However, very little information is available about the optimization of photochemical decomposition acetamiprid illuminated with TiO_2_/Fe_3_O_4_/SiO_2_ composite. Therefore, in the current study, applicability of TiO_2_/Fe_3_O_4_/SiO_2_ nanocomposite as an efficient photo catalyst for the degradation of acetamiprid and a novel test for effluent toxicity assessment using *Pseudomonas aeruginosa* were evaluated.

## Materials and methods

### Chemicals and media

Analytical acetamiprid toxin was purchased from Sigma-Aldrich Co. with a purity of 98.5%, while its technical form was purchased from Ariashimi Co, Zahedan with a purity of 97%. Acetic acid, ethanol 99.9%, FeCl_3_, FeCl_2_, tetra ethyl ortho silicate 95%, tetra-*n*-butyl lorthotitanate, ammonium solution, Resazurin powder, agar muller hinton, broth nutrient, KH_2_PO_4_, K_2_HPO_4_, glucose, sodium acetate, dimethyl sulfur oxide (DMSO), *n*-amyl alcohol, HCl–phthalate buffer, and sodium bicarbonate were purchased from Sigma-Aldrich Co.

### Microorganism

*Pseudomonas aeruginosa* BCRC 11864 strain (ATCC™ 27853) was purchased from Microbiology Laboratory, Islamic Azad University Qom.Iran. In order to prepare a fresh culture and activate the bacteria, Agar Muller–Hinton culture medium (34 g/L) was employed.

### Synthesis of Fe_3_O_4_ nanoparticles

To synthesize magnetic nanoparticles Fe_3_O_4_ using co-precipitation method. Firstly, 11.68 g of FeCl_3_·6H_2_O and 4.31 g of FeCl_2_·6H_2_O were dissolved in 200 mL of distilled water for 30 min and stirred at 85 °C. Next, the solution obtained was added to 1.8 L of distilled water and was stirred for at 85 °C for 24 h. In order to prevent oxidation, concurrent magnetic injection of nitrogen gas and bubbling was performed. Thereafter, 20 mL of ammonium solution with 30% W/V was added drop-wise to the solution. Its supernatant was removed by a separator funnel (decanter) and following four washes with distilled water and ethanol, the final magnetic product was obtained (Lopez et al. 2010; Hakami et al. 2012; Zhang et al. 2013; Tian et al. 2014).

### Synthesis of Fe_3_O_4_/SiO_2_/TiO_2_ nanoparticles

Synthesis of Fe_3_O_4_/SiO_2_/TiO_2_ nanocomposite (NPs) was done using sol–gel methods. Before synthesis of titanium dioxide with Fe_3_O_4_, an internal layer (SiO_2_) was used between the TiO_2_ coating, while magnetic compounds were applied for better TiO_2_ coating and preventing iron dissolution. For this purpose, Fe_3_O_4_ nanoparticles were dispersed in 200 mL distilled water containing TEOS under ultrasonic conditions for 20 min. After that, for separation of nanoparticles from each other, there were four washing stages by water and ethanol. For coating Fe_3_O_4_/SiO_2_ nanoparticles with TiO_2_, first 30 mL of acetic acid was added to an iron and silica containing reactor and stirred. Next, a mixture of tetra-*n*-butyl lorthotitanate solution (volume of 15 mL), acetic acid 30 mL, and ethanol 30 mL was prepared. The compound obtained was added to the reactor drop-wise very slowly. The resulting solution underwent ultrasound for 30 min, after which it was kept still for 2 h. In order to dry the nanoparticles, calcination was performed using an electric furnace at 800 °C for 2 h (Wang et al. 2010; Hakami et al. 2012; Shi et al. 2012; Zhan et al. 2014).

### Characteristics of nanocomposite

The surface morphology of the fabricated core–shell Fe_3_O_4_/SiO_2_/TiO_2_ was characterised by SEM (Vega/Tescan-Lmu) analysis after the samples were coated with a thin layer of gold to prevent charge problems and to enhance the resolution. Vibrating Sample Magnetometer studies (VSM) were carried out to study the effect of phase transformations on the magnetic properties of the nanoparticles. The variation of magnetization versus the applied magnetic field for the nanoparticles measured at room temperature.

### Experimental design and statistical analysis

In this study, response surface methodology has been used for the modelling (Sahoo and Gupta 2012; Lee and Hamid 2015; Mansouriieh et al. 2015) of the acetamiprid degradation. The photocatalytic experiments were designed based on CCD model for variables such as; contact time (10–120 min), acetamiprid initial concentration (1–40 mg/L), dose of nanoparticles (0.1–1 g/L), and pH (3–12), Table 1. For data analysis, Design Expert. Ver 7 was used.

**Table 1:** The levels and ranges of variables central composite statistical experiment design

Factor	Name	Low actual	High actual	Low coded	High coded	Mean	Std. dev
A	pH	5.25	9.75	−1.000	1.000	7.500	2.012
B	Contact time (min)	37.50	92.50	−1.000	1.000	65.000	24.597
C	Acetamipraid (mg/L)	10.75	30.25	−1.000	1.000	20.500	8.721
D	Dose of NP^a^ (mg/L)	325.00	775.00	−1.000	1.000	550.000	201.246

^a^ Nanoparticles

### Preparation of reactor

Photocatalytic experiments were performed in a glass reactor with a reflective wall with a volume of 3 L (height 27 cm, width of 10.53 cm) equipped with UV lamp within the range of 251–257 nm, with the peak of 254 nm and a power of 125 W. In each stage of the experiment, 2 L of the solution of the toxin and the nanoparticles synthesized with concentrations of interest was poured into the reactor. Thereafter, the resulting mixture was stirred by a mechanical stirrer.

### Analytical methods

Over the course of the experiment, sampling was conducted according to the design. Once samples were taken from the reactor, centrifugation was conducted at 1000*g* for 5 min to separate the nanoparticles from the solution better. Next, using a magnet with an intensity of 8 T, nanoparticles inside the samples were precipitated and samples filtered using a syringe equipped with a 0.22 μm filter. The concentration of acetamiprid was measured using HPLC (model Agilent Technologies 1260 Infinity) equipped with UV lamp detector and the following specifications were used: C18 column, length and diameter of the column were 4.6 mm × 250 mm, wavelength was 260 nm, mobile phase containing a mixture of deionized water and methanol had a volume ratio of (50/50), the matrix inside the bed column contained pure spherical particles of silica gel, the ambient temperature during the measurement was 25 ± 2 °C, and the volume of injection sample = 20 µL. pH, temperature, and ORP were obtained using a multiparametric probe (HQ40d). The degree of mineralization of the Acetamiprid was ascertained by measuring the chemical oxygen demand (COD) according to standard methods (Ensano et al. 2017). Also, the by-products resulting from decomposition of acetamiprid were analyzed by gas chromatography–mass (GC–MS) (Wang and Shih 2016).

### Measurement of UV lamp intensity

In order to measure the intensity of UV lamp (125 W) with average pressure in the reactor, radiometer device Hanger ECL-X, specifically designed for measuring the intensity of UV-C radiation range lamps, was used. In order to compare the intensity of lamp in a typical reactor and reflective wall reactor, it was controlled and measured at a distance half of the reactor length within different time intervals. The intensity of UV radiation was determined using Eq. 1

1 $${\text{Intensity of UV radiation }}\;({\text{w}}/{\text{cm}}^{2} ) = {\text{T}}\; \times \;{\text{L}}$$

where: T = time (s), $${\text{L}} = {\text{light intensity}}\;({\text{w}}/{\text{s}}\,{\text{cm}}^{2} )$$

### Toxicity assessment test based on RR methods

In order to determine the extent of reduction of Resazurin dye using dehydrogenase enzyme, first broth nutrient culture medium with a concentration of 1.6 g/L was enriched with KH_2_PO_4_ (1.64 g/L), K_2_HPO_4_ (2.64 g/L), glucose (0.2 g/L), and sodium acetate (0.2 g/L). Next, 2750 µL of the enriched broth nutrient culture medium was poured into a test tube. A total of 250 µL DMSO, 1000 µL of bacterial suspension, and 1000 µL of Resazurin solution (100 mg/L) were added to the solution.

In the next stage, 1000 µL of the toxin containing solution was added with specific concentrations (diluted with distilled water). Thereafter, it was incubated in darkness at 28 °C. Following 30 min of contact time, 10 mL of *n*-amyl alcohol solution and 1 mL of HCl–phthalate 0.05 M buffer were added to each test tube. Following the mixing and centrifugation (1000*g* in 5 min), the top alcohol layer was transferred to a sink of microplate containing 2–3 g sodium bicarbonate. Once these materials were stirred slowly, the extent of Resazurin reduction was determined through the extent of absorption at the wavelength of 610 nm using UV/Vis spectrophotometer device.

In the presence of active and alive bacteria, the activity of dehydrogenase enzyme caused reduction of Resazurin and its conversion to Resorufin, whereby the color of the solution changed from blue to pink. Inactive bacteria do not develop any changes in Resazurin and its color remains blue (Liu 2006; Tizzard et al. 2006; Zare et al. 2015). The percentage of reduction in the growth was obtained using Eq. 2.

2 $${\text{Diminished activity of dehydrogenase enzyme in Resazurin conversion}}\;\% = {{\left( {{\text{A}} - {\text{B}}} \right)} \mathord{\left/ {\vphantom {{\left( {{\text{A}} - {\text{B}}} \right)} {{\text{A}} \times 1 0 0}}} \right. \kern-0pt} {{\text{A}} \times 1 0 0}}$$

where: A, the extent of activity of dehydrogenase enzyme in the control sampleand B, the extent of activity of dehydrogenase enzyme in the main sample.

### Toxicity assessment test based on CFU methods

Firstly, one loop of *P. aeruginosa* was added to 25 mL of broth culture medium to prepare bacterial suspension. The turbidity developed in the suspension had a density of about 3 × 10^8^ cells/mL. Thereafter, dilution was performed until its optical density was equal to a solution 1 McFarland by this culture medium. After that, its turbidity was measured by a spectrophotometer device at wavelength of 625 nm (Toolabi et al. 2011). In the next stage, 100 µL of bacteria containing suspension was inoculated on a plate containing the culture medium and acetamiprid toxin at different concentrations. Finally, using Eq. 3, the growth inhibition percentage was determined.

3 $$\% {\text{Growth inhibition}}\;{ = }\;{{{\text{A}} - {\text{B}}} \mathord{\left/ {\vphantom {{{\text{A}} - {\text{B}}} {\text{A}}}} \right. \kern-0pt} {\text{A}}} \times 100$$

where: A, the number of colonies of the control sample and B, the number of colonies of the inoculated sample.

## Results

### Scanning electron microscopy

**Fig. 1:**
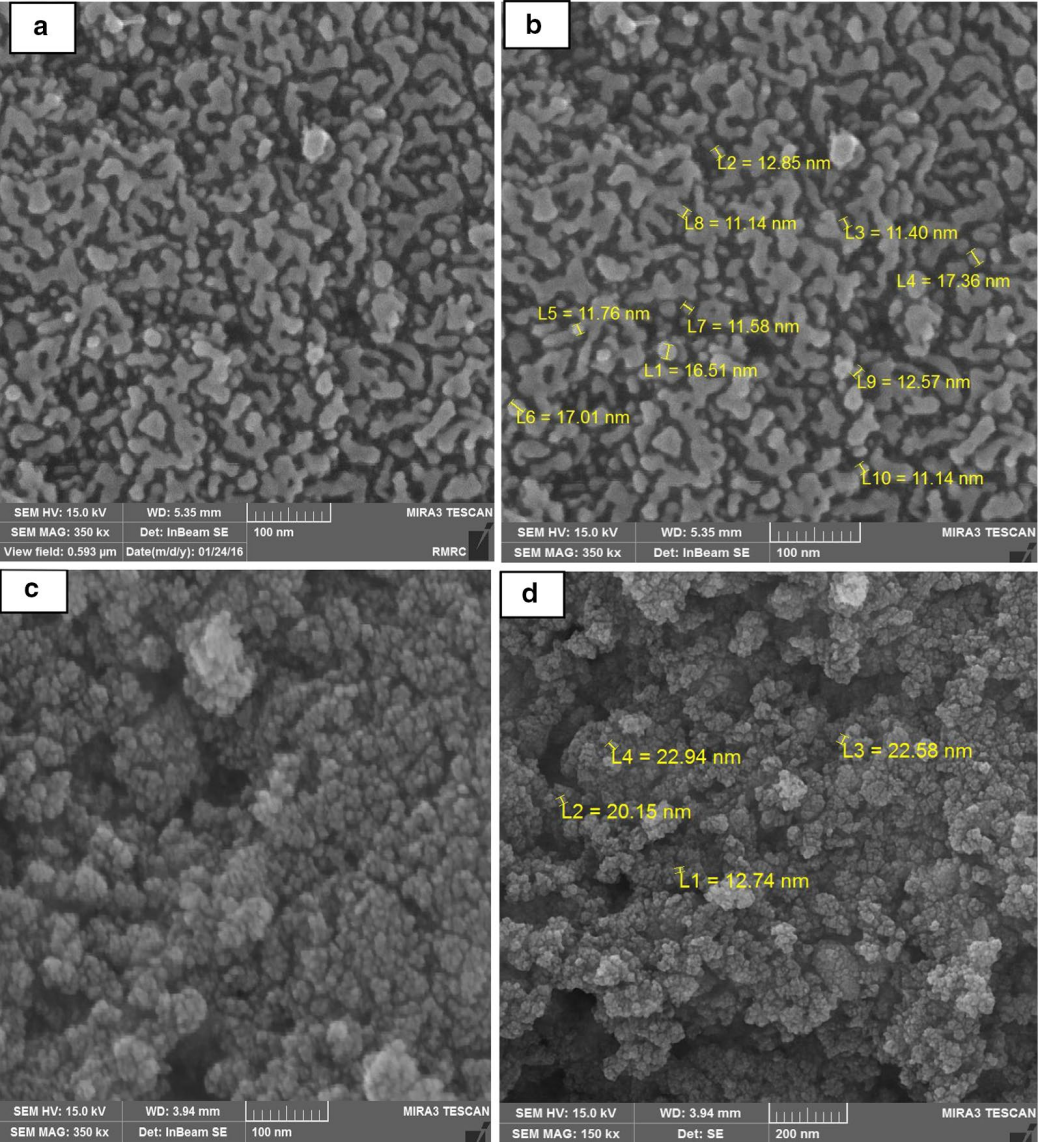
SEM images of **a**, **b** Fe_3_O_4_, **c**, **d** Fe_3_O_4_/SiO_2_/TiO_2_

Figure 1 shows the SEM images at higher magnification and the size distributions of particles were in the range of 100–200 nm, also it gave clear idea about the particle separation, we can see that the particles are separated smoothly and not highly affected by agglomeration.

### Vibrating sample magnetometer

**Fig. 2:**
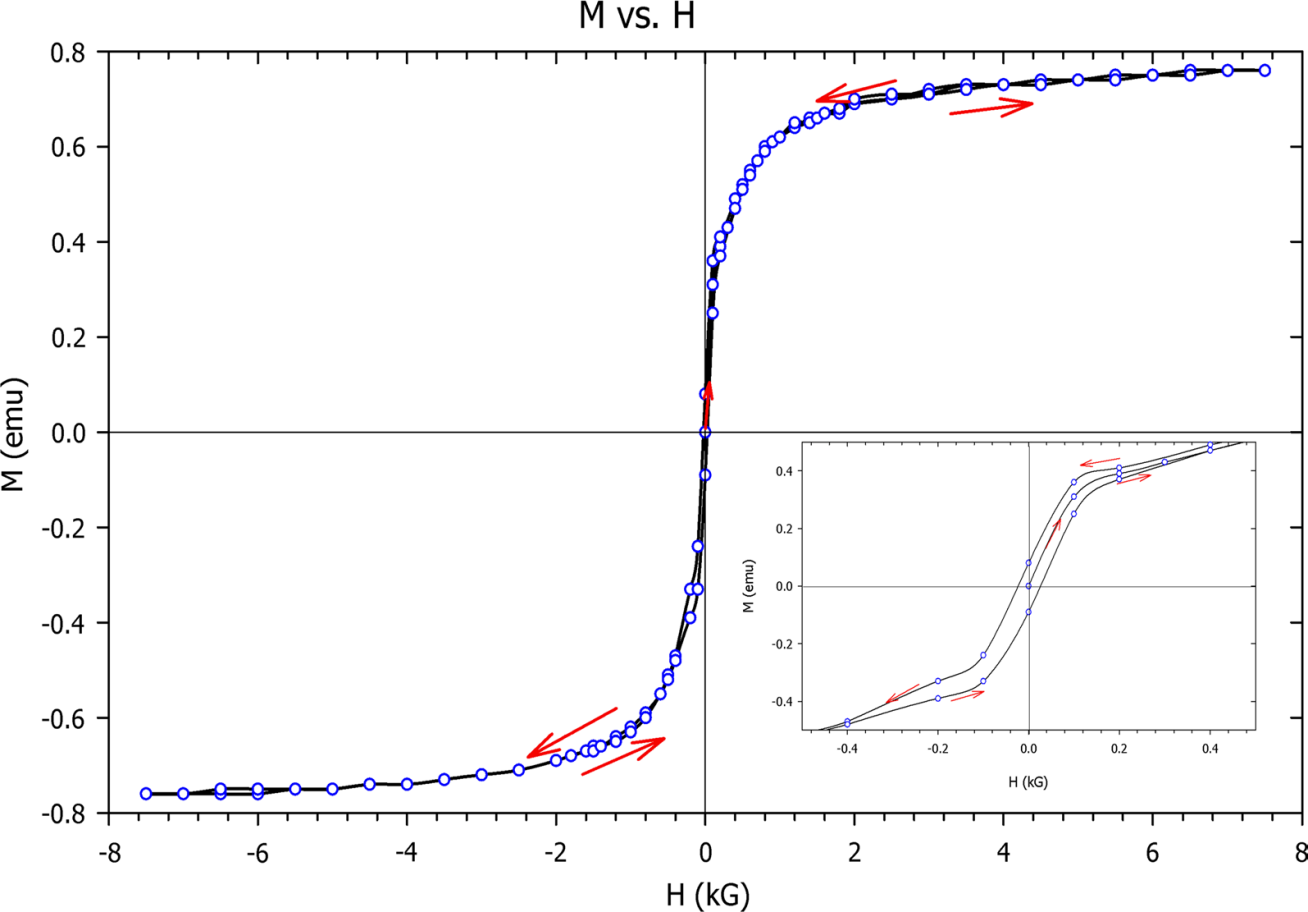
VSM result of naked Fe_3_O_4_/SiO_2_/TiO_2_

VSM was used to evaluate magnetization of the NPs as a function of an applied external magnetic field (H) between −8 and 8 T with a resolution of 10^−4^ emu. Based on the obtained VSM curve at room temperature, magnetic behaviours of the NPs can be analyzed. The zero magnetic remanence (when H is zero) and the hysteretic loop feature indicates that the NPs are super paramagnetic. The dependences of magnetic momentum of NPs on magnetic field are shown Fig. 2.

### The effect of variables

**Fig. 3:**
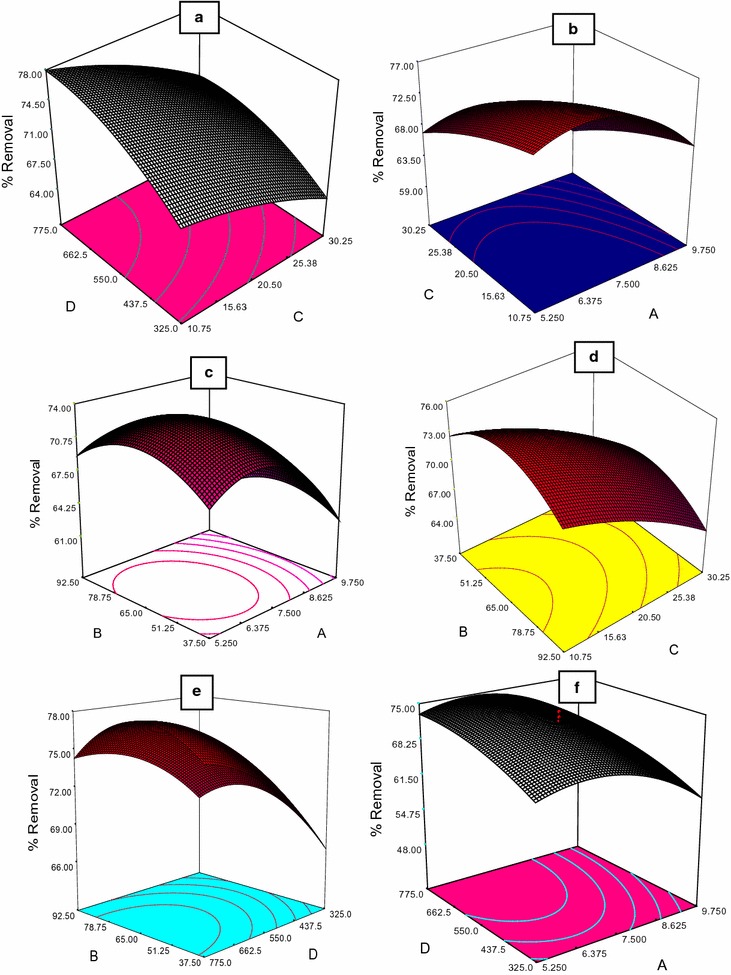
Three-dimensional response removal of acetamiprid with interactions between factors. **a** The effect of dose of nanoparticles (mg/L) and concentrations of acetamiprid (mg/L). **b** The effect of concentrations of acetamiprid (mg/L) and pH. **c** The effect of contact time (min) and pH. **d** The effect of contact time (min) and concentrations of acetamiprid (mg/L). **e** The effect of contact time (min) and dose of nanoparticles (mg/L). **f** The effect of dose of nanoparticles (mg/L) and pH

In this study, three-dimensional response and contour plot in removal of acetamiprid with interactions among factors such as pH, contact time, concentrations of acetamiprid and dose of nanoparticles were studied Fig. 3 and Table 2. It was observed that maximum removal efficiency of acetamiprid is (greater than 73%) when pH is near 6.5, contact time is between 65 and 73 min, initial concentrations of acetamiprid and dose of nanoparticles were 20.55 and 550 mg/L, respectively.

**Table 2:** Results of the experimental runs designed according to the CCD

Run	Factor A: pH	Factor B: contact time (min)	Factor C: concentration of acetamiprid (mg/L)	Factor D: dosage of nanoparticles (mg/L)	Removal efficiency (%)	Predicted value
1	5.250	92.50	10.75	325.0	64.80	64.80
2	9.750	37.50	10.75	325.0	59.42	59.41
3	5.250	92.50	10.75	325.0	67.51	67.53
4	9.750	65.00	10.75	325.0	59.85	59.84
5	5.250	92.50	30.25	325.0	58.80	58.79
6	9.750	37.50	30.25	325.0	52.85	52.85
7	5.250	92.50	30.25	325.0	60.69	60.69
8	9.750	65.00	30.25	325.0	52.44	52.45
9	5.250	10.00	10.75	775.0	73.61	73.61
10	9.750	92.50	10.75	775.0	68.12	68.12
11	5.250	92.50	10.75	775.0	73.00	72.99
12	9.750	65.00	10.75	775.0	65.20	65.21
13	5.250	37.50	30.25	775.0	65.97	65.97
14	9.750	65.00	30.25	775.0	59.95	59.93
15	5.250	37.50	30.25	775.0	64.50	64.52
16	9.750	37.50	30.25	775.0	56.19	56.18
17	3.000	65.00	20.50	550.0	61.75	61.74
18	12.00	92.50	20.50	550.0	48.00	48.01
19	7.500	65.00	20.50	550.0	61.58	61.59
20	7.500	120.00	20.50	550.0	60.59	60.57
21	7.500	65.00	1.000	550.0	76.55	76.54
22	7.500	37.50	40.00	550.0	61.50	61.50
23	7.500	37.50	20.50	100.0	59.48	59.48
24	7.500	65.00	20.50	1000.	72.02	72.02
25	7.500	65.00	20.50	550.0	71.55	72.63
26	7.500	37.50	20.50	550.0	73.00	72.63
27	7.500	65.00	20.50	550.0	70.54	72.63
28	7.500	92.50	20.50	550.0	72.80	72.63
29	7.500	65.00	20.50	550.0	74.43	72.63
30	7.50	65.00	20.50	550.0	73.46	72.63

### Modeling

According to various models (linear, two-factor interaction, quadratic and cubic) and their subsequent ANOVA, the acetamiprid removal suitably illustrated by the quadratic model (R^2^ = 0.9939). The predicted values of the response obtained from the model were sufficiently correlated to the observed values. The following quadratic equation describes the acetamiprid removal efficiency as a function of the variables in terms of cod factors, Eq. 4.

4 $$\begin{aligned} \;{\text{Y}}\,=\,& 7 2. 6 3 \; - 3. 4 3 2\; \times \; {\text{A}} - 0. 2 5 50 \; \times \; {\text{B }} - 3. 7 5 9 \; \times \; {\text{C 3}}. 1 3 6 \; \times \; {\text{D}}\; - 0. 5 7 3 7 \; \times \;{\text{A}}\; \times \; {\text{B}}\; - 0. 1 3 7 5 \\ & \times \;{\text{A}}\; - 0.0 2 3 7 5 \; \times \; {\text{A}} \times {\text{D}} - 0. 2 100 \; \times \; {\text{B}}\; \times \;{\text{C}}\; - 0. 8 3 6 3\; \times \; {\text{B}}\; \times \; {\text{D}}\; - 0. 40 7 5\; \times \; {\text{C}}\; \times \; {\text{D}}\; - 4. 4 3 9 \\ & \times \;{\text{A}}^{ 2} - 2. 8 8 7\; \times \; {\text{B}}^{ 2} - 0. 90 1 7\; \times \; {\text{C}}^{ 2} - 1. 7 20 \; \times \; {\text{D}}^{ 2} \\ \end{aligned}$$

where: Y represent acetamiprid removal (%) A, B, C and D are the coded values of pH, contact time, initials concentration of acetamiprid and dose of nanoparticles, respectively. The experimental data for acetamiprid removal were statistically analyzed by analysis of variance.

### By-products formation

According to the results, 24 compounds with a detection quality of over 50% were detected based on molecular weight. By-products with molecular weight from 98.11 to 444.113 atomic mass unit (AMU) were detected within different retention times of 2.232 until 28.113 min. The clearest peaks were related to Nonadecane, Octadecane, Cyclohexasiloxane–dodecamethyl, Cyclotrisiloxane–hexamethyl, and Heptane compounds, whose detection quality was over 90%. The major peak at the time of 19.334 min was related to Pentasiloxane–dodecamethyl compound, whose detection quality was obtained to be 47%.

### The effect of reactor type on intensity of UV radiation

UV radiation was generated both inside the reactor with a reflective wall and a reactor with an aluminum wall. According to the results obtained, the extent of intensities of radiation emitted in the reflective reactor and aluminum reactor were 180 and 125 W/cm^2^, respectively. This difference had been due to greater reflection of radiation from the reactor walls, which was 1.44 times more than the one observed in the aluminum reactor under similar conditions. Therefore, removal efficiency of acetamiprid was observed due to greater presence of radiation, causing rapid electron excitation off the catalyst surface by UV radiation. Other properties of these reactors include generation of less heat in the solution and being economical, such that when the reflective reactor was used. The maximum measured temperature of the solution was 32 °C, and under the same conditions, the temperature observed in the aluminum reactor was above 43 °C.

### Effect concentration of acetamiprid in RR and CFU

In order to determine the inactive rate of *P. aeruginosa* BCRC 11864, effective concentration (EC) parameter was used. Accordingly, inhibition level of 10–100% was investigated, Table 3. EC_50_ (mg/L) related to RR and CFU methods were 1.950 and 2.050, respectively. Furthermore, 100% mortality (mg/L) using the two methods stood at 4.180 and 4.280, respectively.

**Table 3:** The result of acetamiprid effect concentration in Resazurin reduction and colony forming unit

Parameters	Type of test	Value	p value
EC_10_ (mg/L)	RR	0.895	<0.05
	CFU	0.901	<0.05
EC_20_ (mg/L)	RR	1.180	<0.05
	CFU	1.020	<0.05
EC_30_ (mg/L)	RR	1.380	<0.05
	CFU	1.410	<0.05
EC_40_ (mg/L)	RR	1.745	<0.05
	CFU	1.884	<0.05
EC_50_ (mg/L)	RR	1.950	<0.05
	CFU	2.050	<0.05
EC_60_ (mg/L)	RR	2.160	<0.05
	CFU	2.250	<0.05
EC_70_ (mg/L)	RR	2.468	<0.05
	CFU	2.578	<0.05
EC_80_ (mg/L)	RR	2.753	<0.05
	CFU	2.800	<0.05
EC90 (mg/L)	RR	3.110	<0.05
	CFU	3.230	<0.05
NOEC^a^ (mg/L)	RR	0.521	<0.05
	CFU	0.630	<0.05
100% mortality (mg/L)	RR	4.180	<0.05
	CFU	4.280	<0.05

^a^ No observed effect concentration

## Discussion

The Fe_3_O_4_/SiO_2_/TiO_2_ nanoparticles were successfully synthesized by sol–gel methods. The SEM image clearly indicates that the as-synthesized Fe_3_O_4_/SiO_2_/TiO_2_ nanoparticles are composed of spheres having uniform features. According to the results in the current study, The ANOVA of the second order quadratic polynomial model for the responses show that the models are significant. The Model F-value of 175.85 implies the model is significant, Table 4. There is only a 0.01% chance that a model F value this large could occur due to noise. Values of Prob > F less than 0.0500 indicate model terms are significant (Lee and Hamid 2015; Dong and Sartaj 2016). In their study, It was observed that maximum removal efficiency was when pH near 6.5 and contact time between 65 and 73 min. Minimum removal efficiency when pH near 9. It was also observed that at lower pH values, the removal of acetamiprid was more than alkaline state. As a result, more hydrolysis of acetamiprid occurred at solutions containing hydrogen. According to the results Fig. 3 contact time was an effective parameter in decomposition of acetamiprid toxin, such that with the increase in contact time from 10 to 65 min, the extent of acetamiprid decomposition grew from 38 to 75%. After this, the removal efficiency saw only minor changes, but at contact time of 70 min, it became constant. This is due to greater electron excitation off the catalyst’s surface, production of more active radicals, and the possibility of greater physical collision of the catalyst’s active agents with acetamiprid, providing enough time for collision and decomposition of acetamiprid.

**Table 4:** ANOVA for response surface quadratic model: analysis of variance table (partial sum of squares—Type III)

Source	Sum of squares	df	Mean square	F value	*p* value	Prob > F
Model	1581	14	112.9	175.85	<0.0001	Significant
A-pH	282.6	1	282.6	440.2	<0.0001	–
B-contact time	1.561	1	1.561	2.430	0.1398	–
C-Concentration of acetamiprid	339.2	1	339.2	528.2	<0.0001	–
D-dose of N.P	236.0	1	236.0	367.5	<0.0001	–
AB	5.267	1	5.267	8.203	0.01183	–
AC	0.3025	1	0.3025	0.4711	0.5030	–
AD	0.009025	1	0.009025	0.01406	0.9072	–
BC	0.7056	1	0.7056	1.099	0.3111	–
BD	11.19	1	11.19	17.43	0.06001	–
CD	2.657	1	2.657	4.138	<0.0001	–
A^2^	540.5	1	540.5	841.8	<0.0001	–
B^2^	228.6	1	228.6	356.0	<0.0001	–
C^2^	22.30	1	22.30	34.73	<0.0001	–
D^2^	81.18	1	81.18	126.4	<0.0001	–
Residual	9.632	15	0.6421	–	–	–
Lack of fit	0.002383	10	0.0002383	0.0001238	1.0000	Not significant
Pure error	9.629	5	1.926	–	–	–
Cor total	1590	29	–	–	–	–

According to the results Fig. 3 and through plotting the single-factor curve, it was found that with increase in the concentration of acetamiprid toxin, removal efficiency declined, such that with increase in the concentration from 1 to 40 mg/L, under the same experimental conditions, the removal efficiency decreased from 76.5 to 61.5%. This difference is due to the fact that when acetamiprid is decomposed, the higher its initial concentration, the greater the number of molecules and hydrolyzed particles in the solution. Furthermore, high-energy electrons of the catalyst and the free radicals remained at a certain level of the solution and could not reach all acetamiprid molecules to decompose them.

According to the results from plotting single-factor curve in CCD, with the increase in concentration of TiO_2_/Fe_3_O_4_/SiO_2_ in the experimental environment, removal efficiency of acetamiprid and COD increased. However, at the concentration of 325 mg/L of nanoparticles, their removal level was 61 and 79% respectively. When the concentration of the nanoparticles increased to 550 mg/L, their removal level reached 76 and 95%, respectively. This is due to the fact that when the concentration of nanoparticles increases in the solution of the experiment and is accompanied by UV radiation, the extent of excitation of e^−^ and generation of h^+^ increases in the catalyst surface. When in contact with water molecules, h^+^ produces hydroxyl radicals and H^+^ ions, and e^−^ gets into the reaction with dissolved oxygen for formation of superoxide ions (O_2_·). Next, its reaction with water molecules cause generation of hydroxide ions and peroxide radicals (OOH·). These peroxide radicals are mixed with H^+^ ions and produce OH·. These hydroxyl radicals collide with acetamiprid and decompose it (Cao et al. 2013; Rodriguez et al. 2013; Verma and Sillanpaa 2015).

According to the results in the current study, the toxicity of acetamiprid is evident at low concentrations for *P. aeruginosa* BCRC 11864 bacteria. Furthermore, there is a direct and positive relationship between the inhibition percentage of the activity of dehydrogenase enzyme of the bacteria and inhibition percentage of the colonies that formed (R^2^ = 0.987). In the study by Zare et al. (2016), RR method and its comparison with CFU of *Pseudomonas, Enterobacter,* and *Bacillus* bacteria were evaluated in determining the toxicity of wastewater containing heavy metals. In their study, it was found that there is a linear and direct relationship between the bacterial enzymatic activity and colony count in determination of toxicity. Therefore, the results of this study were in line with the conducted study.

According to the results in the current study, a strong relationship was seen between the extent of decomposition of acetamiprid and reduction of toxicity where greater the extent of acetamiprid decomposition, lower the extent of toxicity in both toxicity determination methods of RR and CFU. When the wastewater produced by the reactor was allowed to have contact for 150 min, the extent of growth inhibition of *P. aeruginosa* BCRC 11864 bacteria reached 2% using both the mentioned methods. These results suggest relatively good decomposition of acetamiprid in the solution by the TiO_2_/Fe_3_O_4_/SiO_2_/UV photocatalytic process and output of products whose toxicity is far lower than that of the acetamiprid. According to the results presented in Table 5, there is a significant relationship between the extent of absorption of Resazurin and oxidation reduction potential (ORP). In aerobic respiration, ORP level is positive, and typically an ORP above +50 mV represents aerobic conditions and bacterial activity. Lower the extent of absorption of Resazurin greater is the extent of conversion of Resazurin to Resorufin by *P. aeruginosa* BCRC 11864 bacteria, and the level of ORP is also higher.

**Table 5:** Results of the experimental ORP, COD and absorption of Resazurin

Run	Effluent of acetamiprid (mg/L)	Absorption of Resazurin	ORP (mv)	COD initial (mg/L)	COD residuals (mg/L)
1	3.784	0.066	221.6	48	6.72
2	4.362	0.069	221.0	48	7.68
3	3.492	0.063	222.6	48	5.28
4	4.316	0.068	221.8	48	7.00
5	12.461	0.110	214.0	142	28.40
6	14.262	0.118	213.2	142	29.00
7	11.891	0.101	215.0	142	27.20
8	14.386	0.119	209.8	142	29.00
9	2.847	0.040	232.0	48	4.68
10	3.427	0.064	224.2	48	5.30
11	2.902	0.043	232.7	48	4.80
12	3.741	0.061	223.9	48	6.55
13	10.294	0.095	215.4	142	29.10
14	12.115	0.108	214.1	142	30.00
15	10.738	0.099	215.2	142	29.4
16	13.252	0.115	210.0	142	31.10
17	7.841	0.085	215.7	96	16.32
18	10.661	0.097	215.3	96	17.34
19	7.176	0.083	216.2	96	15.20
20	8.079	0.087	215.8	96	15.50
21	0.214	0.013	243.0	4.2	0.21
22	15.391	0.121	207.0	168	33.60
23	8.306	0.088	216.0	96	17.30
24	5.731	0.080	216.3	96	14.56
25	5.422	0.071	220.5	96	14.79
26	5.432	0.076	220.0	96	15.00
27	5.424	0.071	220.5	96	14.80
28	5.422	0.070	220.5	96	15.00
29	5.446	0.079	220.4	96	14.86
30	5.440	0.077	220.5	96	15.00

This is due to higher presence of *P. aeruginosa* aerobic bacteria in the culture environment containing acetamiprid toxin, which managed to remain alive and have enzymatic activity. Furthermore, there is an indirect and linear significant relationship (p < 0.05) between the extent of conversion of Resazurin to Resorufin and the level of residual chemical oxygen demand (COD_r_). The greater the extent of conversion of Resazurin to Resorufin by the bacteria, the higher is the extent of COD decomposition and lower the residue in the aqueous solutions.

Moreover, in the current study, a simple method, rapid, and economical based on the activity of dehydrogenase enzyme was optimized for determining the effluent toxicity assessment of aqueous solutions, so that the extent of reduction of Resazurin was in proportion to the extent of activity of *P. aeruginosa* BCRC 11864 bacteria. Also, a scientific and significant relationship was demonstrated between the oxidation reduction potential and colony forming unit with reduction of Resazurin. This had not been proven so far with regard to acetamiprid pesticide.

## Abbreviations

RSM: response surface methodology
CCD: central composite design
CFU: colony forming unit
RR: Resazurin reduction
ORP: oxidation reduction potential
AMU: atomic mass unit
